# Presence of virulent *Edwardsiella tarda* in farmed nile tilapia and striped catfish

**DOI:** 10.1186/s12866-025-04232-9

**Published:** 2025-09-05

**Authors:** Lamiaa A. Okasha, Enas A. H. Farag, Rasha M. H. Sayed-ElAhl, Ahmed H. Sherif

**Affiliations:** 1 Bacteriology Unit, Agriculture Research Center ARC, Animal Health Research Institute AHRI, Kafrelsheikh, 12619 Egypt; 2 Department of Pharmacology, Agriculture Research Center ARC, Animal Health Research Institute AHRI, Benha, 12619 Egypt; 3 Department of Mycology, Animal Health Research Institute AHRI, Agriculture Research Center ARC, Giza, 12619 Egypt; 4 Fish Diseases Department, Agriculture Research Center ARC, Animal Health Research Institute AHRI, Kafrelsheikh, 12619 Egypt

**Keywords:** *Pangasianodon hypothalamus*, *Oreochromis niloticus*, *Edwardsiella tarda*, Antibiotic residue

## Abstract

The production of striped catfish *(Pangasianodon hypothalamus)* has increased worldwide; recently, it was farmed with Nile tilapia in polyculture farms. Polyculture systems and water temperature (25℃ and 33℃) could affect *Edwardsiella tarda* infection, antibiotic efficacy, and residues. Moribund fishes were collected from three Farms 1–3: Farm 1 (monoculture, Nile tilapia), Farm 2 (monoculture, striped catfish), and Farm 3 (polyculture). Four *E. tarda*, LAMSH1, and LAMAH2-4 were isolated, whereas LAMAH3 was isolated from both fish spp., where striped catfish were highly susceptible to infection. The obtained *E. tarda*, which was isolated from striped catfish, has a significantly lower LD_50_ than those retrieved from Nile tilapia, and co-infection occurred only in striped catfish on Farm 3. The infection was screened and confirmed by *gyrB1* gene presence while detecting the *cds1*,* pvsA*, and *qseC* genes indicated virulence. All isolates were sensitive to ciprofloxacin and florfenicol but showed resistance to a high number of other antibiotics, resulting in high multi-drug resistant (MDR) indices exceeding 0.2, except for strain LAMAH4, which had an index of 0.18.

Analyses of farms water revealed high ammonia compounds total ammonia nitrogen (TAN), unionized ammonia (NH_3_), nitrite (NO_2_), and nitrate (NO_3_) in Farm 2 (monoculture, striped catfish), and the recorded significantly higher concentrations were 2.75, 0.29, 0.24, and 2.01 mg/L, respectively, which were compared with Farm 1 and Farm 3. In the indoor experiment, at high water temperatures (33 °C), Nile tilapia and striped catfish had a high mortality rate and re-isolation of *E. tarda* (10–20%) compared to those exposed to low water temperatures (25 °C). These observations were concurrent with low antibiotic residues in their hepatic tissues. Despite water temperature, Nile tilapia showed higher ciprofloxacin residues than striped catfish.

The study concluded that striped catfish are more susceptible to the bacteria *E. tarda* compared to Nile tilapia, particularly in polyculture farms, which resulted in a higher infection rate. Both Nile tilapia and striped catfish exposed to elevated water temperatures exhibited increased vulnerability to bacterial infections. Additionally, these fish showed a high re-isolation rate of *E. tarda* while having low ciprofloxacin residues in their hepatic tissues.

**Supplementary Information:**

The online version contains supplementary material available at 10.1186/s12866-025-04232-9.

## Introduction

More than 156 million tons of fish products are supplied to the global market year-based, and aquaculture production covers about 46% of human consumption [1]. Within Africa, Egyptian production formed approximately 70% of aquaculture crops, with about 1.57 million tons in 2021 [2]. The Egyptian fish farms are mainly located in the northern Nile River delta, around and south of the freshwater lakes; local markets consume these productions with low exports. Kafrelsheikh governorate has the highest number of fish farms and hatcheries, making about half of national production [3].

Nile tilapia *(Oreochromis niloticus)* form about 67% of the Egyptian aquaculture harvest, mainly produced in semi-intensive earthen ponds (3 to 5 acres in area) and fed commercial ration (crude protein, 25%). In Egypt, fish farms depend on polyculture systems Nile tilapia, African catfish *(Clarias gariepinus)*, thin lip mullet *(Mugil capito)*, and gray mullet *(Mugil cephalus)* [4]. However, the expansion of fish production uses large amounts of formulated diets, wastes, and uneaten feed turned into free ammonia and phosphorus, especially in warm-water aquaculture [5]. Usually, the intensification of aquatic animal production is correlated with infectious disease outbreaks such as bacterial diseases, which raise the emergence of antibiotic-resistant bacteria [6, 7]. *Edwardsiellosis* is the most common disease in catfish farms, and it could infect both farmed and wild fish, causing high financial loss. Moreover, the causative agent is *E. tarda*, a highly pathogenic bacterium belonging to *Enterobacteriaceae* with a wide range of hosts and countries; many virulence genes increase its survivability and pathogenicity [8]. This bacterium is a Gram-negative bacillus and has five pathogenic species: *E. tarda*,* E. ictaluri*,* E. piscicida*,* E. hoshinae*, and *E. tarda* [9–11]. *Edwardsiella* isolates showed many phenotypic and interspecific diversity recovered from different countries and host kinds; therefore, rapid, accurate, and specific confirmation of the causative agents is a fundamental step for controlling disease and epizootiological investigations [12]. The infection of *E. tarda* has been found in *C. gariepinus* [13], *P. pangasius* [14], and Nile tilapia [15].

This study discusses the impact of introducing a new member to Egyptian aquaculture striped catfish *(Pangasianodon hypothalamus)*, such as a rise of *E. tarda* infection in the polyculture of Nile tilapia and striped catfish. Caged Nile tilapia, which fed untreated marine fish offal, were infected with *Vibrio parahaemolyticus*, which usually prevailed in marine fish species [16]. Previous studies stated that the introduction of common carp *(Cyprinus carpio)* resulted in the occurrence of *Lernaea cyprinacea* infestation in freshwater fish in Egyptian farms and hatcheries [17, 18].

To combat *Edwardsiellosis*, veterinarians and fish farmers use different antibiotics with different doses and schedules; the abuse of antibiotics applications is a usual practice in low- and middle-income countries, resulting in antibiotic resistance requesting alternative antimicrobial agents [19–23]. Many *Edwardsilla spp.*, which were recovered from various fish species, have developed high levels of resistance against a wide range of antibiotics [24], with a potential spread becoming a major concern for human health. Antibiotic withdrawal period mainly controlled by water temperature [25]. In this study, Nile tilapia and striped catfish were subjected to two different temperature degrees (25 °C and 33 °C), and antibiotic residues were determined after a challenge test and treatment with ciprofloxacin.

This work aims to isolate *Edwardsiella tarda* from Nile tilapia and striped catfish reared in polyculture and monoculture fish farms. Also, retrieved bacteria were identified and confirmed using molecular techniques. An experiment was conducted to investigate the impact of temperatures of rearing water on *E. tarda* infection rate and re-isolation rate as well as response to ciprofloxacin treatment and its residue in the liver tissues of Nile tilapia and striped catfish.

## Materials & methods

### Fish farms and samples collection

During the production season (April to November 2024), moribund Nile tilapia *(Oreochromis niloticus)* and striped catfish *(Pangasianodon hypothalamus)* were randomly collected from three fish farms encountered mortalities (10–50 deaths per day); the fish pond is earthen-pond about three acres in area and 1 m in depth. Farms 1–3 are designed as follows: Farm 1 has a monoculture with 25,000 Nile tilapia per acre, Farm 2 is a monoculture with 30,000 striped catfish per acre, and Farm 3 is a polyculture with 25,000 Nile tilapia and 10,000 striped catfish per acre. Fish farms are located at village Tolmpat 7 in north Egypt.

Fish samples were collected from Farm 1, Farm 2, and Farm 3. Each farm provided 60 Nile tilapia and 60 striped catfish. The collected fish were placed into aerated, clear plastic bags filled with water containing a tranquilizing agent, tricaine methanesulfonate (MS-222, Syndel, Canada), at a concentration of 40 mg/L [26, 27]. The fish were immediately transported to the Animal Health Research Institute laboratory for further analysis.

### Water samples analyses

At farm sites, water temperature and salinity were determined (YSI Environmental, EC300), dissolved oxygen (DO) (Aqualytic, OX24), and pH (Thermo Orion, 420 A). Three water samples, one from each pond, were parallelly collected with fish samples from each farm at a depth of 0.5 m of the fish pond using aseptic one-liter plastic bottles to avoid bacterial contamination. Then, the bottles were transported into the ice box and sent to the laboratory. At the laboratory, Animal Health Research Institute, water samples were examined for total ammonia nitrogen (TAN), unionized ammonia (NH_3_), nitrite (NO_2_), and nitrate (NO_3_) using UV/visible spectrophotometer, Thermo-Spectronic 300.

### Microbiological examination of the bacterial isolates

The examination of fish samples was done to determine the occurrence of bacterial infections in farmed fish samples, following the recommendations of Woo and Bruno [28].

#### The preliminary isolation

Fish organs, brain, kidney, spleen, and liver were swabbed into tryptic soy broth (Difco, Detroit, USA) for 24 h at 30 °C. A loopful from each bacterial strain was re-cultured onto tryptic soy agar (TSA) (Difco, Detroit, USA) supplemented with 5% sheep blood for 24 h at 30 °C. The most prevailed bacterial colonies (similar size and shape) on TSA were harvested and re-cultivated onto selective media (Salmonella-Shigella agar) (Difco, Detroit, USA) phenotypic profiles of *E. tarda* were according to the instruction of Bergey [29]. Then, they were preserved in tryptic soy broth with an equal amount of glycerol solution (30%) at −80 °C. Further identification was performed with Gram-stain and API 20E (bio-Merieux) [30, 31].

#### Molecular identification

DNA of *Edwardsiella* spp were extracted and examined for *gyrB1* gene and virulence genes: *cds1* (chondroitinase), *qseC* sensor protein, and *pvsA* (vibrioferrin synthesis). The DNA extraction was done with the PathoGene-spin™ DNA Extraction Kit (iNtRON Biotechnology, Seongnam, Korea). Then, the polymerase chain reaction (PCR) kits (Bioline, Meridian Life Science, UK) were used to replicate target sequences. The PCR products were run into agarose gel 1% to separate distinct bands using electrophoresis (Applichem, Germany, GmbH), and the bands were photographed using a gel documentation system (Alpha Innotech, Biometra). All primers and gene amplification used in this study are listed in (supplementary).

#### Sequence of bacterial DNA

Amount of 50 ng/µl the bacterial DNA was preserved (−20 ℃) for *E. tarda gyrB1* sequencing, the amplicons (bands) were cut off the gel and purified (QIAquick extraction kits, Qiagen, Germany), the target gene was sequenced (Big Dye terminator v3.1 kit, Life Technologies, Applied Biosystems, Foster City, CA, USA) using the ABI 3730xl DNA-sequencer (Applied Biosystems™, USA). The raw sequences were manually edited (sequence alignment editor, BioEdit v. 7.2.5) [32] before being submitted to the GenBank database to access the accession numbers. The Neighbor-Joining phylogenetic tree was developed to display the results using the MEGA X program [33].

#### Antimicrobial sensitivity analyses

The method of disc diffusion was performed in triplicates to detect the sensitivity and resistance of the four *E. tarda* isolates to different antibiotics that are used in aquaculture, such as tetracycline (TE) 30 µg, ciprofloxacin (CIP) 5 µg, trimethoprim/sulfamethoxazole (SXT) 1.25/23.75 µg, erythromycin (E) 15 µg, florfenicol (F) 30 µg, gentamicin (GEN) 30 µg, amoxicillin 10 µg, kanamycin (K) 30 µg, cefotaxime (CTX) 30 µg, ampicillin (AMP) 10 µg, and streptomycin (S) 30 µg, All antibiotic discs obtained from Oxoid™. Isolates were enriched into TSB for 12 h, then cultured onto Mueller-Hinton agar (Oxoid™), and antimicrobial discs were organized on the plates. Disc antibiotic concentrations, the diameter of the inhibition zones was determined, and the results were interpreted according to the standards of the Clinical Laboratory Standards Institute (CLSI 2010) as resistant, intermediate, and sensitive [34]. The multidrug resistance (MDR) of *E. tarda* strains was calculated using the following equation:

$$\:\mathbf{M}\mathbf{D}\mathbf{R}\:=\frac{\:\mathbf{X}}{\mathbf{Y}}$$

Where x is the number of antibiotics in which the isolates were resisted, and y is the tested antibiotics, MDR greater than 0.2 means that the strain is multi-resistant [35].

### Lethal median dose and pathogenicity testing

The LD_50_ of the *E. tarda* strains was calculated using steps provided by Reed and Muench [36]. Four hundred Nile tilapia and four hundred striped catfish with a mean weight of (70.8 ± 0.3 g and 92 ± 1.1 g, respectively) were purchased from a private fish hatchery hatchery in Kafrelsheikh governorate tranquilized with MS-222 at a concentration of 40 mg/L of transporting water. Each *E. tarda* isolates serial tenfold dilutions (24 h-old culture); 10^1^, 10^2^, 10^3^, 10^4^, 10^5^, 10^6^, 10^7^, 10^8^, 10^9^, and 10^10^ CFU/mL. The bacterial counts were adjusted using McFarland standard and re-cultivated on TSA to ensure the accuracy of the count. A 100 µl suspension of each dilution was injected intraperitoneally into ten Nile tilapia and ten striped catfish. Mortalities were observed for fourteen successive days post-challenge.

### Experimental infection, antibiotic treatment, and antibiotic residues

One hundred and forty healthy Nile tilapia and one hundred and forty striped catfish with a mean weight of (70.8 ± 0.3 g and 92 ± 1.1 g, respectively) were purchased from a private fish hatchery and transported to the wet laboratory at Animal Health Research Institute, Kafrelsheikh. Fishes were subdivided equally into two groups (120 individuals for each fish species) and acclimated in 24 glass aquaria 40 × 40 × 50 cm (10 fish/aquarium) at two different water temperatures, 25℃ and 33℃. Twenty Nile tilapia and twenty striped catfish were kept as controls.

To examine the pathogenicity of *E. tarda*, 30 fish (Nile tilapia and striped catfish) were intraperitoneally injected with 0.1 mL of *E. tarda* solution containing LD_50_ of LAMSH1 and LAMAH2-4 strains obtained from this study, the bacteria dose was adjusted to LD_50_ using the McFarland scale in phosphate buffer saline and re-cultivated in TSA for the accuracy of the bacterial number. Also, a negative control group was formed by injecting ten fish with pure normal saline (0.65 mg/L), according to Boijink et al. [25]. Each dead fish was counted for 14 days if *E. tarda* was re-isolated and confirmed using PCR following the scheme in the above Sect. (2.3.2.). The mortality rates (MR) were estimated using the following equation:

$$\mathrm{MR}\%\;=\;\frac{\mathrm{deaths}\;\mathrm{in}\;\mathrm a\;\mathrm{specific}\;\mathrm{period}}{\mathrm{total}\;\mathrm{population}}\;\times100$$

- Antibiotic treatment and residues: Fish were treated with ciprofloxacin (Cip) the trade name is ciprofar (500 mg) manufactured by Pharco pharmaceuticals (Batch No.: 21515/2012), Amirya-Alexandria, Egypt.
- Commercial feed (pellets) was soaked in water and then blended with 5% (w/w) gelatin (Nutri-B-Gel, produced by Canal Aqua Cure, Port-Said, Egypt) and mixed into a paste. The Cip was added at a dose of 10 mg/kg b.w./day [37], past was allowed to dry, then cut into 2 mm-thick pellets. Fish feeds were offered once a day for 10 successive days at a rate of 1% of fish b.w.
- Water parameters: temperature was 25℃ and 33℃ in the two groups aquaria, while values of water salinity, DO, and pH were remained at 0.5 g/L, 5.45 mg/L, and 7.3, respectively, during the experimental period. Ammonia compounds remained below 0.2 mg/L by replacing one third of aquaria water with clean dechlorinated tap water (with the same water temperature).
- The residues of Cip were detected in the hepatic tissues using high-performance liquid chromatography (HPLC). Briefly, tissues were thawed and homogenized (IKA-WERKE ULTRA-TURRAX), adding 25 ml of acetonitrile. Samples were centrifuged (Eppendorf Centrifuge 5810 R) at 6000×g for 10 min. The extraction liquid was separated into a flask at 40 °C until it was almost dried using N2 gas, then filtrated into a disposable syringe (0.45 μm filter) and directly injected into the HPLC [38].

### Statistical analyses

The impacts of *E. tarda* on Nile tilapia and striped catfish survivability, bacterial re-isolation, and antibiotic residues were statistically determined by comparing the means of the obtained data using one-way ANOVA test, SPSS software version 22. P-values of the collected data determined using Duncan’s various range, if *P*≤ 0.05 they were statistically significant. Results showed as mean±standard error (SE).

##  Results

In Table 1, the infection rate *E. tarda* infection rate in Farm 3 (polyculture: Nile tilapia and striped catfish) was 35/60 and 60/60, respectively, while it was 5/60 and 20/60 in monoculture farms Nile tilapia (Farm 1) and striped catfish (Farm 2), respectively, regardless of bacterial strain. The strain LAMSH1 was only isolated from striped catfish (Farm 3); meanwhile, LAMAH3 was only isolated from fish on Farm 3, moribund Nile tilapia 35/60 and striped catfish 40/60, while LAMAH4 and LAMAH2 were isolated from both Farm 1 and Farm 2. From the obtained LD_50_, striped catfish were more vulnerable to infection as lower LD_50_ values caused high mortality compared to Nile tilapia. Also, co-infection occurred in striped catfish in polyculture Farm 3.

**Table 1:** Number of naturally infected fish, virulence genes, and median lethal doses of *E. tarda* strains

Items	Fish no.	LAMSH1	LAMAH2	LAMAH3	LAMAH4
		gyrB1	gyrB1, cds1, pvsA	gyrB1, cds1, qseC	gyrB1, qseC
Farm 1 Nile tilapia	60	-	-	-	5
Farm 2 Striped catfish	60	-	20	-	-
Farm 3 polyculture	60 + 60	60	-	35*&40**	-
LD_50_ (CFU/mL) Nile tilapia		3.37 × 10^5^	3.1 × 10^5^	2.95 × 10^5^	3.09 × 10^5^
LD_50_ (CFU/mL) Striped cat fish		0.7 × 10^5^	2.82 × 10^4^	1.78 × 10^4^	3.81 × 10^4^

Farm polyculture, fish population formed of Nile tilapia and striped catfish. LD_50_, median lethal dose. *cds1, *chondroitinase, *pvsA;* vibrioferrin synthesis, and *qseC, *sensor protein

*Nile tilapia

**striped catfish

**Fig. 1:**
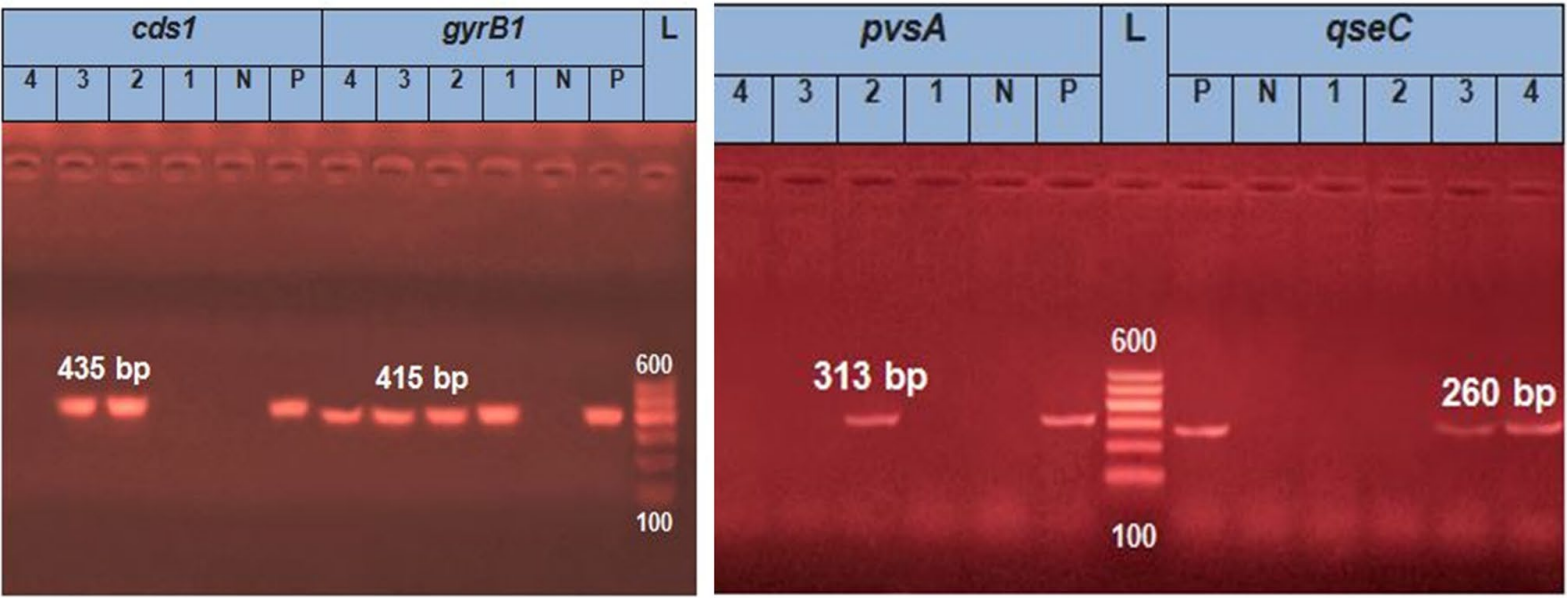
DNA bands on electrophoresis gel of *Edwardsiella tarda gyrB1* and virulence genes: *cds1;* chondroitinase, *pvsA;* vibrioferrin synthesis, and *qseC;* sensor protein. P, positive, N; negative, L; ladder, *E. tarda* strains 1; LAMSH1, 2; LAMAH2, 3; LAMAH3, 4; LAMAH4

In Fig. 1; Table 1, different patterns of *gyrB1* and virulence genes *cds1*, *pvsA*, and *qseC* were detected in the four isolates. The strain LAMSH1 did not harbor the three virulent genes under investigation, and co-infected striped catfish with LAMAH3 harbored *cds1* and *qseC.* Meanwhile, LAMAH3 was the only isolated strain from Nile tilapia (monoculture) and contained *qseC*,* cds1*, while *pvsA* was recorded only in LAMAH2, which was isolated from striped catfish (monoculture).

**Fig. 2:**
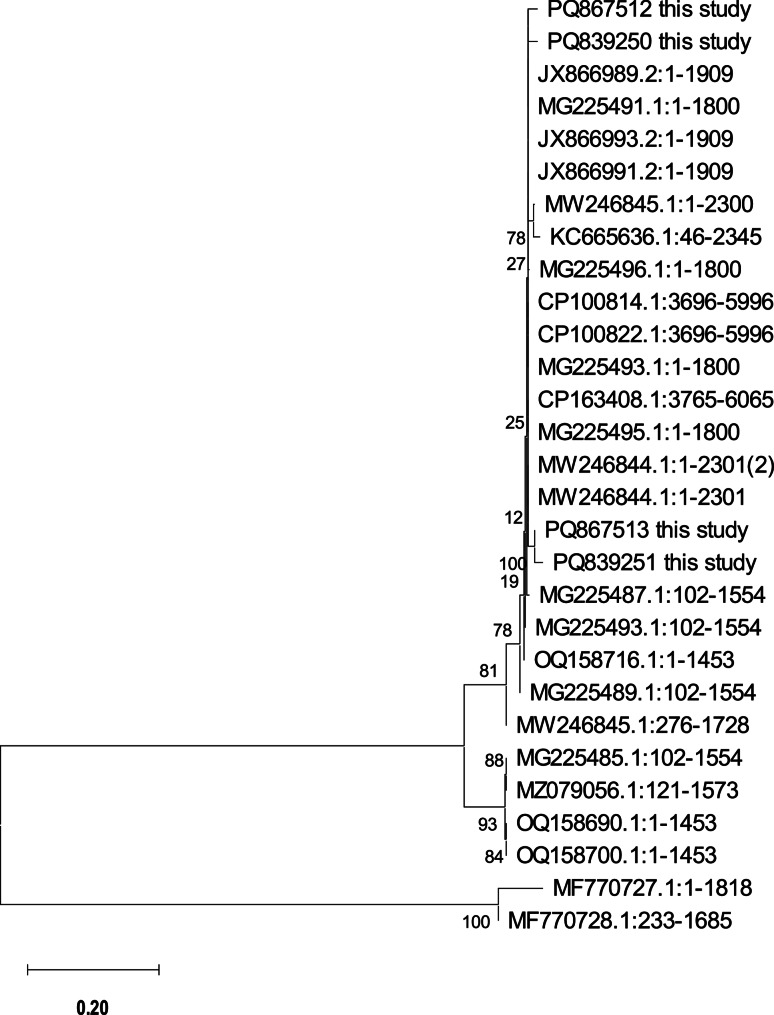
Sequences of *gyrB1* gene of the four *E. tarda* isolates

Four *E. tarda* strains LAMSH1 and LAMAH2-4, were sequenced for *gyrB1* and submitted in the NCBI under accessions numbers PQ839250, PQ839251, PQ867513, and PQ867512, respectively. In Fig. 2, the phylogenetic tree revealed that LAMAH2 and LAMAH3 were genetically related, whereas LAMSH1 and LAMAH4 were also near and harbor low virulence genes with low LD_50_ values.

In Table 2, the four isolates of *E. tarda* were examined for antibiotic sensitivity. All strains were sensitive to ciprofloxacin and florfenicol. Also, it was noticed that strains were resistant to high numbers of antibiotics, and the MDR indices of LAMSH1, LAMAH2, and LAMAH3 were greater than 0.2 and were 0.55, 0.27, and 0.55, respectively, whereas LAMAH4 was 0.18 (Table 3).

**Table 2:** Antibiogram of the isolated *Edwardsiella tarda* strains

No.	Bacteria Strain (*N* = 4)	S (%)	IM (%)	*R* (%)
1	Tetracycline (TE) 30 µg	-	-	LAMSH1 LAMAH2-4
2	Trimethoprim (SXT) 1.25 µg Sulfamethoxazole 23.75 µg	-	-	LAMSH1 LAMAH2-4
3	Ciprofloxacin (CIP) 5 µg	LAMSH1 LAMAH2-4	-	-
4	Florfenicol (F) 30 µg	LAMSH1 LAMAH2-4	-	-
5	Erythromycin (E) 15 µg	LAMAH 2,4	LAMSH1 LAMAH3	-
6	Gentamycin (GEN) 30 µg	-	LAMAH2,4	LAMSH1 LAMAH3
7	Amoxicillin (AML) 30 µg	-	LAMSH1 LAMAH2-4	-
8	Ampicillin (AMP) 10 µg	-	LAMSH1 LAMAH2-4	-
9	Kanamycin (K) 30 µg	-	LAMAH2,4	LAMSH1 LAMAH3
10	Cefotaxime (CTX) 30 µg	-	LAMAH2,4	LAMSH1 LAMAH3
11	Streptomycin (S) 30 µg	-	LAMAH4	LAMSH1 LAMAH2,3

S Sensitive, *IM* Intermediate, R Resistant

**Table 3:** Data on multidrug resistant index of *E. tarda* strains

No.	Bacteria Strain	Antibiotics resistant	MDR index
1	LAMSH1	TE, SXT, S, GEN, K, CTX	0.55
2	LAMAH2	TE, SXT, S	0.27
3	LAMAH3	TE, SXT, S, GEN, K, CTX	0.55
4	LAMAH4	TE, SXT	0.18

*MDR* Multidrug resistant genes, *TE* Tetracycline, *CIP* Ciprofloxacin, *SXT* trimethoprim/sulfamethoxazole, *S* Streptomycin, *GEN* Gentamycin, *K* Kanamycin and, *CTX* Cefotaxime

From Table 4, it was clear that there were no statistical differences in water physical parameters temperature, pH, and DO between farms under investigation. The water of fish farms was brackish, where salinity was significantly lower in Farm 2 (1,27 g/L) compared to the water of Farm 2 (1,97 g/L) and Farm 3 (1,93 g/L); however, these values were.

Water chemical analyses revealed that ammonia compounds TAN, NH3, NO2, and NO3 in Farm 2 (monoculture, striped catfish) were significantly higher at 2.75, 0.29, 0.24, and 2.01 mg/L, respectively, compared with Farm 1 (monoculture, Nile tilapia) and polyculture Farm 3.

**Table 4:** Water analyses of the investigated fish farms

Items	Temp ℃	pH	DO mg/L	Salinity g/L	TAN mg/L	NH_3_ mg/L	NO_2_mg/L	NO_3 _mg/L
Farm 1 Monoculture Nile tilapia	25.73 ± 0.23	8.03 ± 0.12	4.7 ± 0.15	1.27^**B**^ ± 0.07	0.52^**C**^ ± 0.01	0.034^**B**^ ± 0.008	0.04^**B**^ ± 0.01	0.43^**B**^ ± 0.2
Farm 2 monoculture striped catfish	25.57 ± 0.23	8.24 ± 0.14	4.89 ± 0.35	1.97^**A**^ ± 0.07	2.75^**A**^ ± 0.12	0.29^**A**^ ± 0.09	0.24^**A**^ ± 0.01	2.01^**A**^ ± 0.38
Farm 3 Polyculture	25.87 ± 0.2	8.4 ± 0.09	4.68 ± 0.52	1.93^**A**^ ± 0.08	1.65^**B**^ ± 0.1	0.183^**AB**^ ± 0.05	0.05^**B**^ ± 0.02	0.52^**B**^ ± 0.2

Different letter in the same column indicates significant difference at *P* ≤ 0.05

*Temp* water temperature, *pH* Hydrogen ion, *DO* Dissolved oxygen, *TAN* Total ammonia, *NH*_*3*_ unionized ammonia, *NO*_*2*_ Nitrite, and *NO*_*3*_ nitrate

Nile tilapia (Table 5) and striped catfish (Table 6) were challenged with four *E. tarda* isolates in two different water temperatures, 25 °C and 33 °C, with the obtained LD_50_. Then, fish were treated with sensitive antibiotic ciprofloxacin and re-isolation of *E. tarda* attempts and antibiotic residues were performed.

At a high temperature (33 °C) water, Nile tilapia showed a higher MR% and RI%, about 10–20%, over those at a low water temperature (25 °C) concurrently with low antibiotic residues in their hepatic tissues. Striped catfish showed similar findings as Nile tilapia except for the strain LAMSH1 differences of water temperature did not affect the MR; however, the re-isolation rate was increased by 10% over lower temperatures (25 °C). Regardless of fish spp, antibiotic residues were decreased in the hepatic tissues of those exposed to a high temperature of 33 °C; Nile tilapia showed higher ciprofloxacin residues than striped catfish regardless of water temperature.

**Table 5:** Experimental nile tilapia challenged against isolated *E. Tarda.* (*n* = 30)

Water temperature	27 °C	25 °C					33 °C				
Nile tilapia	LD_50_	MR (%)		RI (%)		AR (µg)	MR (%)		RI (%)		AR (µg)
		UT	T	UT	T		UT	T	UT	T	
Control	Saline 0.65%	10	-	-	-	-	-	-	-	-	-
LAMSH1	3.37 × 10^5^	30	10	60	20	7.04	40	20	80	30	6.6
LAMAH2	3.1 × 10^5^	40	20	60	30	7.3	60	30	70	50	6.73
LAMAH3	2.95 × 10^5^	50	20	70	30	7.15	60	40	80	50	6.55
LAMAH4	3.09 × 10^5^	30	20	60	30	7.24	40	30	70	40	6.4

LD_50_ median lethal dose, *MR* Mortality rate, *RI* Re-isolation, *AR* Antibiotic residues, *UT* Untreated, *T* Treated with antibiotic

**Table 6:** Experimental striped catfish challenged against isolated *E. tarda*. (*n* = 10)

Water temperature	27 °C	25 °C					33 °C				
Striped catfish	LD_50_	MR (%)		RI (%)		AR (µg)	MR (%)		RI (%)		AR (µg)
		UT	T	UT	T		UT	T	UT	T	
Control	Saline 0.65%	-	-	-	-	-	-	-	-	-	-
LAMSH1	0.7 × 10^5^	50	40	80	70	6.3	50	40	80	60	5.08
LAMAH2	2.82 × 10^4^	70	50	90	90	6.16	80	60	100	90	5.1
LAMAH3	1.78 × 10^4^	70	50	100	80	6.09	80	60	100	90	5.2
LAMAH4	3.81 × 10^4^	60	40	90	70	6.2	80	60	100	80	5.32

LD_50_ median lethal dose, *MR* Mortality rate, *RI* Re-isolation, *AR* Antibiotic residues, *UT* Untreated, *T* Treated with antibiotic

## Discussion

In this work, four *E. tarda* were isolated and identified in three monoculture and polyculture fish farms, experienced mortalities of about 10–50 deaths daily with a high isolation rate Nile tilapia (35/60) and striped catfish (60/60). *Edwardseilla* spp. occurred in a wide range of aquatic animals, mainly those exposed to high water temperatures and an overload of organic matter [39]. In addition, susceptible fish may infected with *E. tarda* via gills, skin, or oral route [40]; for example, striped catfish experienced *Edwardsiellosis* among several diseases’ challenges and infections outbreaks as many freshwater fishes [41, 42], the *Edwardsiellosis/*emphysematous putrefactive disease is a septicaemic recorded in some fish farms associated with mass mortality in various populations and ages of striped catfish; the disease is usually observed in tilapia, carp, eel, catfish, mullet, and flounder [41, 43].

Different phenotypic variations and interspecific diversity between *E. tarda* isolates were retrieved from different fish species, so disease control and epidemiological investigations need rapid and validated detection of the isolates [12, 44]. In this study, the identification of *E. tarda* isolates was confirmed by the presence *of gyrB1* and virulence genes *cds1*, *pvsA*, and *qseC*; also, the isolates harbored different virulence gene patterns. Previous studies stated that *E. tarda* possesses several virulence-related and toxin secretion system-related genes such as *gyrB1*, *edw*I, *cds1*, *qseC*, and *pvsA* through which bacterium could survive within macrophages and detoxifying organs (liver and the kidney) as well as it could infect a wide range of aquatic animals [45]. In this work, the obtained LD_50_ indicated that striped catfish were more vulnerable to *E. tarda* infection, possessing low LD_50_ values. Interestingly, healthy striped catfish were intramuscularly injected with a sub-lethal dose of 1.77 × 10^7^ cells *E. tarda*/fish showed *Edwardsiellosis* signs while MR% reached 42% at 48 h post-injection [46].

In this work, the isolated *E. tarda* were sensitive to ciprofloxacin and florfenicol but were resistant to many antibiotics. Also, MDR was higher than 0.2, indicating a multi-drug-resistant bacteria. Accordingly, in polyculture systems, Zhang et al. [47] found that antibiotic-resistance bacteria (ARB) prevailed in the fish gut, mucosal skin, and gill filaments in farms of hybrid grouper (*Epinephelus fuscoguttatus*♀ × *E. lanceolatus*♂*)*,* Gracilaria bailinae*, and *Litopenaeus vannamei* with different stocking combinations, researchers suggested that differences in antibiotic resistance were derived by polyculture system cause changes in bacterial communities. Conversely, findings were claimed by Huang et al. [48], who claimed that ARB is more prevalent in fish reared in monoculture farms than those in integrated multitrophic aquaculture (IMTA) systems. In contrast to our findings, Yuan et al. [49] stated that ARB was more isolated from bullfrog ponds than polyculture ponds due to the more frequent use of antimicrobial treatments with seven categories of commonly used antibiotics (e.g., aminoglycosides, beta-lactams, sulfonamides, tetracyclines). Besides aquaculture systems, other factors control the abundance of ARG; the hospital sewage water in a polluted duckweed farm and ARG had the same resistance pattern determined of wastewater as high strains numbers of antibiotic resistance *Aeromonas* were recovered [50].

In this work, water examination revealed high TAN, NH_3_, NO_2_, and NO_3_ levels, mainly in Farm 2 (monoculture, striped catfish), suggesting a role in bacterial distribution and prevalence. Similar results with different fish species, Zheng et al. [51] experimented on different farm system models; four different fish: black carp *(Mylopharyngodon piceus)*, largemouth bass *(Micropterus salmoides)*, yellow catfish *(Pelteobagrus fulvidraco)*, and pearl mussel *(Hyriopsis cumingii)* each in a separate pond, every pond was stocked with three of Chinese carps (silver, bighead, and gibel) in fish polyculture or mussel, it was noticed that the bacterial diversity and distribution using 16 S rDNA in the ponds was statistically correlated with ammonia compounds, claiming that aquaculture mode is a factor regulating the microbial community at the genus level. Similar to the results of high ammonia compounds in monoculture striped catfish, largemouth bass and yellow catfish pond had higher water ammonia, nitrite, TN, and TP levels than the pearl mussel pond [52, 53]. In this context, the water exchange rate and accumulation of organic matter could play a role in the prevalence of virulent bacteria that facilitate the formation of a bacterial biofilm as one form of organic soiling on the surface, which could protect bacteria from disinfectants [54–56].

In the indoor experiment, Nile tilapia and striped catfish maintained at a high water temperature of 33 °C showed high MR% and RI%. Accordingly, in the summer season, climate change correlated with alterations in bacterial communities in water, whereas the feed habitat of fish changes, resulting in increasing the abundance of potential pathogens (e.g., the genera *Vibrio*,* Aeromonas*, and *Shewanella*) in fish guts and increase nitrogen wastes in the pond which cause stressful environment to crucian carp [57, 58]. The low ciprofloxacin residues in the hepatic tissues of Nile tilapia and striped catfish, which reared at the high-water temperature of 33 °C in contrast with low water temperature (25 °C), could be explained by the fact that the clearance of antibiotics from fish tissues depends on water temperature and fish metabolism as high temperature accelerates metabolic processes in poikilothermic animals [59]. Accordingly, Salte and Liestol [60] noticed that sulfonamides elimination from muscular tissues of rainbow trout *(Salmo gairdneri)* was controlled by water temperature and salt concentrations, suggesting a withdrawal time of 60 days and 100 days at water temperature over 10 °C and 7–10 °C, respectively, while low clearance of antibiotic in low water temperature could be due to the downregulation of metabolic rate. From the results obtained, consumers who received the ciprofloxacin-treated Nile tilapia or striped catfish were subjected to considerable amounts of antibiotics. Ciprofloxacin and enrofloxacin could resist biodegradation [61], and even wastewater treatment cannot eliminate it [62]. Moreover, ciprofloxacin and enrofloxacin can accumulate in aquatic animals and were identified in several fish kinds [63]. Similarly, in a previous study, ciprofloxacin in water was 70 ng/L accumulated in the fish at a concentration of 7.5 × 10^−6^ µg/g, whereas humans consumed 113 g of fish containing this level, the contents of the human intestinal had a concentration of 2 × 10^−6^ µg/mL suggesting that ciprofloxacin residues are unlikely to be problematic, however resulted in intestinal dysbiosis and antibiotic resistance [64].

In this experiment, the causative agent of fish deaths was confirmed based on the RI of the *E. tarda* isolates rather than recording the clinical and post-mortem signs. Previous studies confirmed that hemorrhagic bacterial diseases have similar clinical and post-mortem signs in addition to *Edwardsiella* spp. share many phenotypic characters [12, 13, 15].

Future studies could spotlight the effect of polluted water (heavy metals and pesticides) on bacterial DNA mutation, decreased antibiotic efficacy, and the impacts of polyculture on fish immunity that made fish more vulnerable to bacterial infection.

## Conclusion

Polyculture striped catfish and Nile tilapia were associated with a high rate of *E. tarda* isolation. Striped catfish were more susceptible to Edwadsiellosis with low LD_50_ values. The monoculture striped catfish was associated with the high ammonia compound in the water. All isolated strains were sensitive to florfenicol and ciprofloxacin with different patterns of virulence-related genes. From the experimental trial, a highwater temperature of 33 °C increases the infection and re-isolation rates with low ciprofloxacin residues in the hepatic tissue regardless of fish kind.

## Supplementary Information

Supplementary Material 1.

## Data Availability

Data are available on request from the corresponding author.
