# Supplementary material for: Presence of virulent Edwardsiella tarda in farmed nile tilapia and striped catfish
Source: BMC Microbiol. 2025 Sep 5;25:572. doi: 10.1186/s12866-025-04232-9 (PMC12412243; doi:10.1186/s12866-025-04232-9)
Supplement: Supplementary file 1 — Supplementary Material 1. [file 12866_2025_4232_MOESM1_ESM.docx]

**Nile tilapia**

**Table (1). Calculation of *E. tarda* LD_50_ by Reed-Muench method. LAMSH1**

| **Dose** | **Log** | **Ob mor** | **Death** | **surv** | **Accumulated** | | | **Mor** | **MR** |
| --- | --- | --- | --- | --- | --- | --- | --- | --- | --- |
|  |  |  |  |  | **death** | **Sur** | **Total** |  |  |
| **0** | 0 | 0/5 | 0 | 5 | 0 | 44 | 44 | 0/45 | 0 |
| **10^1^** | 1 | 1/10 | 1 | 9 | 1 | 39 | 40 | 1/40 | 2.5 |
| **10^2^** | 2 | 1/10 | 1 | 9 | 2 | 30 | 32 | 2/32 | 6.25 |
| **10^3^** | 3 | 3/10 | 3 | 7 | 5 | 21 | 26 | 5/26 | 19.23 |
| **10^4^** | 4 | 3/10 | 3 | 7 | 8 | 14 | 22 | 8/22 | 36.36 |
| **10^5^** | 5 | 8/10 | 8 | 2 | 16 | 7 | 23 | 15/23 | 65.22 |
| **10^6^** | 6 | 8/10 | 8 | 2 | 24 | 5 | 29 | 23/29 | 79.3 |
| **10^7^** | 7 | 8/10 | 8 | 2 | 32 | 3 | 35 | 31/35 | 88.6 |
| **10^8^** | 8 | 9/10 | 9 | 1 | 41 | 1 | 42 | 40/42 | 95.24 |
| **10^9^** | 9 | 10/10 | 10 | 0 | 51 | 0 | 51 | 50/51 | 98.04 |

Note: *0 CFU = control group injected intraperitoneal normal saline 0.065%; Ob. Mor.= observed mortality; Sur. No. = survival number; MR = mortality r ate.

Proportional distance (PD) = (65.22-50) / (65.22-36.36) = 15.22 /30.42 = 0.52737

50% end point = 10^(5+0.52737^ = 10^5.52737^ =3.37 × 10^5^

**Table (2). Calculation of *E. tarda* LD_50_ by Reed-Muench method. LAMAH2**

| **Dose** | **Log** | **Ob mor** | **Death** | **Surv** | **Accumulated** | | | **Mor** | **MR** |
| --- | --- | --- | --- | --- | --- | --- | --- | --- | --- |
|  |  |  |  |  | **Death** | **Sur** | **Total** |  |  |
| **0** | 0 | 0/5 | 0 | 5 | 0 | 41 | 41 | 0/41 | 0 |
| **10^1^** | 1 | 0/10 | 1 | 9 | 1 | 36 | 37 | 1/36 | 2.77 |
| **10^2^** | 2 | 2/10 | 2 | 8 | 3 | 27 | 33 | 3/33 | 9.1 |
| **10^3^** | 3 | 3/10 | 3 | 7 | 6 | 19 | 25 | 6/25 | 24 |
| **10^4^** | 4 | 5/10 | 5 | 5 | 11 | 12 | 23 | 11/23 | 47.8 |
| **10^5^** | 5 | 6/10 | 6 | 4 | 17 | 7 | 24 | 17/24 | 70.8 |
| **10^6^** | 6 | 9/10 | 9 | 1 | 26 | 3 | 29 | 26/29 | 89.66 |
| **10^7^** | 7 | 9/10 | 9 | 1 | 35 | 2 | 37 | 35/37 | 94.6 |
| **10^8^** | 8 | 9/10 | 9 | 1 | 44 | 1 | 45 | 44/45 | 97.8 |
| **10^9^** | 9 | 10/10 | 10 | 0 | 54 | 0 | 54 | 54/54 | 100 |

Note: *0 CFU = control group injected intraperitoneal normal saline 0.065%; Ob. Mor.= observed mortality; Sur. No. = survival number; MR = mortality rate.

Proportional distance (PD) = (70.8-50) / (70.8-47.8) = 20.8 / 30 = 0.49145

50% end point = 10^(5+0.49145)^ = 10^5.49145^= 310,063.= 3.1× 10^5^

**Table (3). Calculation of *E. tarda* LD_50_ by Reed-Muench method. LAMAH3**

| **Dose** | **Log** | **Ob mor** | **Death** | **Surv** | **Accumulated** | | | **Mor** | **MR** |
| --- | --- | --- | --- | --- | --- | --- | --- | --- | --- |
|  |  |  |  |  | **Death** | **Sur** | **Total** |  |  |
| **0** | 0 | 0/5 | 0 | 10 | 0 | 46 | 46 | 0/46 | 0 |
| **10^1^** | 1 | 1/10 | 1 | 9 | 1 | 36 | 37 | 1/37 | 2.7 |
| **10^2^** | 2 | 2/10 | 2 | 8 | 3 | 27 | 30 | 3/30 | 10 |
| **10^3^** | 3 | 2/10 | 2 | 8 | 5 | 19 | 24 | 5/24 | 20.83 |
| **10^4^** | 4 | 5/10 | 5 | 5 | 10 | 11 | 21 | 10/21 | 47.62 |
| **10^5^** | 5 | 5/10 | 5 | 5 | 15 | 6 | 21 | 15/21 | 71.43 |
| **10^6^** | 6 | 9/10 | 9 | 1 | 24 | 1 | 25 | 24/25 | 96 |
| **10^7^** | 7 | 10/10 | 10 | 0 | 34 | 0 | 34 | 34/34 | 100 |
| **10^8^** | 8 | 10/10 | 10 | 0 | 44 | 0 | 44 | 44/34 | 100 |
| **10^9^** | 9 | 10/10 | 10 | 0 | 54 | 0 | 54 | 54/34 | 100 |

Note: *0 CFU = control group injected intraperitoneal normal saline 0.065%; Ob. Mor.= observed mortality; Sur. No. = survival number; MR = mortality rate.

Proportional distance (PD) = (71.43-47.62) / (71.43-20.83) = 23.81 / 50.6 = 0.47055

50% end point = 10^(5+0.^ ^47055)^ = 10^5.47055^ = 295,494 = 2.95 × 10^5^

**Table (4). Calculation of *E. tarda* LD_50_ by Reed-Muench method. LAMAH4**

| **Dose** | **Log** | **Ob mor** | **Death** | **Surv** | **Accumulated** | | | **Mor** | **MR** |
| --- | --- | --- | --- | --- | --- | --- | --- | --- | --- |
|  |  |  |  |  | **Death** | **Sur** | **Total** |  |  |
| **0** | 0 | 0/5 | 0 | 5 | 0 | 45 | 45 | 0/45 | 0 |
| **10^1^** | 1 | 1/10 | 1 | 9 | 1 | 40 | 41 | 1/41 | 2.44 |
| **10^2^** | 2 | 1/10 | 2 | 8 | 3 | 31 | 34 | 3/34 | 8.82 |
| **10^3^** | 3 | 2/10 | 2 | 8 | 5 | 23 | 28 | 5/28 | 17.86 |
| **10^4^** | 4 | 5/10 | 3 | 7 | 8 | 15 | 23 | 8/23 | 34.78 |
| **10^5^** | 5 | 5/10 | 5 | 5 | 13 | 8 | 21 | 13/21 | 61.9 |
| **10^6^** | 6 | 6/10 | 9 | 1 | 22 | 3 | 25 | 22/25 | 88 |
| **10^7^** | 7 | 6/10 | 9 | 1 | 31 | 2 | 33 | 31/33 | 93.9 |
| **10^8^** | 8 | 9/10 | 9 | 1 | 40 | 1 | 41 | 40/41 | 97.6 |
| **10^9^** | 9 | 9/10 | 10 | 0 | 50 | 0 | 50 | 50/50 | 100 |

Note: *0 CFU = control group injected intraperitoneal normal saline 0.065%; Ob. Mor.= observed mortality; Sur. No. = survival number; MR = mortality rate.

Proportional distance (PD) = (88-61.9) / (88-34.78) = 26.1/53.22 = 0.49042

50% end point = 10^(5+0. 49042)^ = 10^5.^ ^0.49042^= 309,328= 3.09 × 10^5^

**Striped catfish**

**Table (1). Calculation of *E. tarda* LD_50_ by Reed-Muench method. LAMSH1**

| **Dose** | **Log** | **Ob mor** | **Death** | **Surv** | **Accumulated** | | | **Mor** | | **MR** |
| --- | --- | --- | --- | --- | --- | --- | --- | --- | --- | --- |
|  |  |  |  |  | **Death** | **Sur** | **Total** |  |  |  |
| **0** | 0 | 0/5 | 0 | 5 | 0 | 31 | 31 | 0 | 31 | 0 |
| **10^1^** | 1 | 1/10 | 1 | 9 | 1 | 26 | 27 | 1 | 27 | 3.7 |
| **10^2^** | 2 | 4/10 | 4 | 6 | 5 | 17 | 22 | 5 | 22 | 22.7 |
| **10^3^** | 3 | 4/10 | 4 | 6 | 9 | 11 | 20 | 9 | 20 | 45 |
| **10^4^** | 4 | 8/10 | 8 | 2 | 17 | 5 | 22 | 17 | 22 | 77.27 |
| **10^5^** | 5 | 9/10 | 9 | 1 | 26 | 3 | 29 | 26 | 29 | 89.7 |
| **10^6^** | 6 | 9/10 | 9 | 1 | 35 | 2 | 37 | 35 | 37 | 94.6 |
| **10^7^** | 7 | 9/10 | 9 | 1 | 44 | 1 | 45 | 44 | 45 | 97.8 |
| **10^8^** | 8 | 10/10 | 10 | 0 | 54 | 0 | 54 | 54 | 54 | 100 |
| **10^9^** | 9 | 10/10 | 10 | 0 | 64 | 0 | 64 | 64 | 64 | 100 |

Note: *0 CFU = control group injected intraperitoneal normal saline 0.065%; Ob. Mor.= observed mortality; Sur. No. = survival number; MR = mortality rate.

Proportional distance (PD) = (77.27-50) / (77.27-45) = 27.27 / 32.27 = 0.845

50% end point = 10^(4+0.^ ^845)^ = 10^4.845^ = 69,984 = 0.7 × 10^5^

**Table (2). Calculation of *E. tarda* LD_50_ by Reed-Muench method. LAMAH2**

| **Dose** | **Log** | **Ob mor** | **Death** | **Surv** | **Accumulated** | | | **Mor** | **MR** |
| --- | --- | --- | --- | --- | --- | --- | --- | --- | --- |
|  |  |  |  |  | **Death** | **Sur** | **Total** |  |  |
| **0** | 0 | 1/5 | 1 | 4 | 1 | 24 | 25 | 1/25 | 4 |
| **10^1^** | 1 | 2/10 | 2 | 8 | 3 | 23 | 26 | 3/26 | 11.54 |
| **10^2^** | 2 | 3/10 | 3 | 7 | 6 | 20 | 26 | 6/26 | 23.1 |
| **10^3^** | 3 | 4/10 | 4 | 6 | 7 | 14 | 21 | 7/21 | 33.33 |
| **10^4^** | 4 | 7/10 | 7 | 3 | 14 | 8 | 22 | 14/22 | 63.64 |
| **10^5^** | 5 | 8/10 | 8 | 2 | 22 | 5 | 27 | 22/27 | 81.5 |
| **10^6^** | 6 | 8/10 | 8 | 2 | 30 | 3 | 33 | 30/33 | 90.9 |
| **10^7^** | 7 | 9/10 | 9 | 1 | 39 | 1 | 40 | 39/40 | 97.5 |
| **10^8^** | 8 | 10/10 | 10 | 0 | 49 | 0 | 49 | 49/49 | 100 |
| **10^9^** | 9 | 10/10 | 10 | 0 | 59 | 0 | 59 | 59/59 | 100 |

Note: *0 CFU = control group injected intraperitoneal normal saline 0.065%; Ob. Mor.= observed mortality; Sur. No. = survival number; MR = mortality rate.

Proportional distance (PD) = (63.64-50) / (63.64-33.33) = 13.64 /30.31 = 0.45

50% end point = 10^(4+0.45)^ = 10^4.45^ = 28183= 2.82 × 10^4^

**Table (3). Calculation of *E. tarda* LD_50_ by Reed-Muench method. Striped cat fish LAMAH3**

| **Dose** | **Log** | **Ob mor** | **Death** | **surv** | **Accumulated** | | | **Mor** | **MR** |
| --- | --- | --- | --- | --- | --- | --- | --- | --- | --- |
|  |  |  |  |  | **death** | **sur** | **total** |  |  |
| **0** | 0 | 1/5 | 1 | 4 | 1 | 39 | 40 | 1/40 | 2.5 |
| **10^1^** | 1 | 2/10 | 2 | 8 | 3 | 35 | 35 | 3/35 | 8.6 |
| **10^2^** | 2 | 3/10 | 3 | 7 | 6 | 27 | 33 | 6/33 | 18.2 |
| **10^3^** | 3 | 4/10 | 4 | 6 | 10 | 19 | 29 | 10/29 | 34.5 |
| **10^4^** | 4 | 6/10 | 6 | 4 | 16 | 13 | 29 | 16/29 | 55.17 |
| **10^5^** | 5 | 6/10 | 6 | 4 | 22 | 9 | 31 | 22/31 | 71 |
| **10^6^** | 6 | 8/10 | 8 | 2 | 30 | 5 | 35 | 30/35 | 85.7 |
| **10^7^** | 7 | 8/10 | 8 | 2 | 38 | 3 | 41 | 38/41 | 92.7 |
| **10^8^** | 8 | 9/10 | 9 | 1 | 47 | 1 | 48 | 47/48 | 97.9 |
| **10^9^** | 9 | 10/10 | 10 | 0 | 57 | 0 | 57 | 57/57 | 100 |

Note: *0 CFU = control group injected intraperitoneal normal saline 0.065%; Ob. Mor.= observed mortality; Sur. No. = survival number; MR = mortality rate.

Proportional distance (PD) = (50-55.17) / (55.17-34.5) = 5.17 /20.67 = 0.25012

50% end point = 10^(4+0.25012)^ = 10^4.25012^ = 1.78 × 10^4^

**Table (4). Calculation of *E. tarda* LD_50_ by Reed-Muench method. LAMAH4**

| **Dose** | **Log** | **Ob mor** | **Death** | **Surv** | **Accumulated** | | | **Mor** | **MR** |
| --- | --- | --- | --- | --- | --- | --- | --- | --- | --- |
|  |  |  |  |  | **Death** | **Sur** | **Total** |  |  |
| **0** | 0 | 0/5 | 0 | 5 | 0 | 29 | 29 | 0/29 | 0 |
| **10^1^** | 1 | 3/10 | 3 | 7 | 3 | 24 | 27 | 3/27 | 11.11 |
| **10^2^** | 2 | 4/10 | 4 | 6 | 7 | 17 | 24 | 7/24 | 29.17 |
| **10^3^** | 3 | 5/10 | 5 | 5 | 12 | 11 | 23 | 12/23 | 47.8 |
| **10^4^** | 4 | 8/10 | 8 | 2 | 20 | 6 | 26 | 20/26 | 76.9 |
| **10^5^** | 5 | 8/10 | 8 | 2 | 28 | 4 | 32 | 28/32 | 87.5 |
| **10^6^** | 6 | 9/10 | 9 | 1 | 37 | 2 | 39 | 37/39 | 94.87 |
| **10^7^** | 7 | 9/10 | 9 | 1 | 46 | 1 | 47 | 46/47 | 97.9 |
| **10^8^** | 8 | 10/10 | 10 | 0 | 56 | 0 | 56 | 56/56 | 100 |
| **10^9^** | 9 | 10/10 | 10 | 0 | 66 | 0 | 66 | 66/66 | 100 |

Note: *0 CFU = control group injected intraperitoneal normal saline 0.065%; Ob. Mor.= observed mortality; Sur. No. = survival number; MR = mortality rate.

Proportional distance (PD) = (76.9-50) / (76.9-47.8) = 16.9 / 29.1 = 0.58075

50% end point = 10^(4+0.8075)^ = 10^4.8075^= 124637= 3.81 × 10^4^
